# Congenital upper auricular detachment: Report of two unusual cases

**DOI:** 10.4103/0970-0358.59298

**Published:** 2009

**Authors:** Pawan Agarwal

**Affiliations:** Plastic Surgery Unit, Department of Surgery, Netaji Subhash Chandra Bose Government Medical College, Jabalpur-482 003, MP, India

**Keywords:** Congenital ear anomaly, partial auricular, detachment, upper auricular, anomalier

## Abstract

Two unusual cases of congenital bilateral ear deformity have been presented. The deformity is characterized by upper auricular detachment on the right side with anotia on the left side in the first case and upper auricular detachment on the left side with normal ear on the right side in the second case. An attempt has been made to correlate the presented deformity with the embryological – foetal development of the auricle. Satisfactory correction can be obtained by repositioning the auricle back in to its normal position.

## INTRODUCTION

A wide variety of congenital auricular malformations are described in literature. These include anotia, microtia, prominent ear, lop ear, cup ear, cryptotia and Stahl's ear. In this article we describe two rare cases of auricular malformation; probably the second case report in the English literature. Although all the congenital auricular malformations are expressions of embryological maldevelopment, as of today, every malformation seen in the ear does not have an embryological explanation. An attempt has been made to co-relate the malformations being presented with the embryologic development of the auricle. Although the cases of lower auricular malformations are common, upper auricular detachment of ear is unusual. Search through the English literature revealed only one case of upper auricular detachment of ear.[1] I. We are reporting two more cases in this article.

## CASE REPORTS

### Case 1

A six- year-old boy presented with congenital anomaly of both auricles. Obstetric history was normal; patient was full term and normally delivered with no history of birth trauma. The pregnancy was also uneventful with no history of any teratogenic exposure.

**Figure 1:**
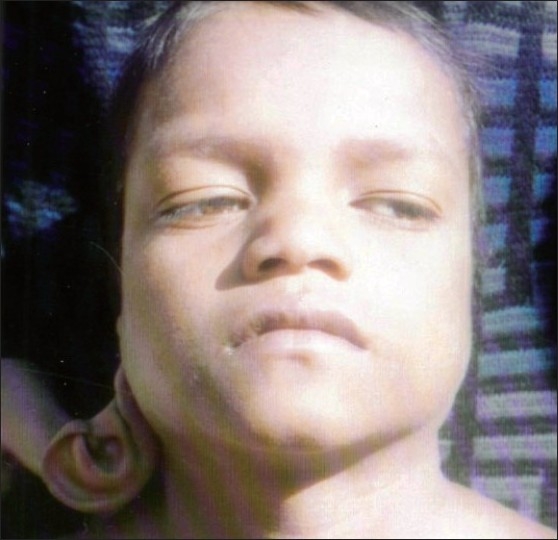
Frontal view showing upper auricular detachment with downward rotation of auricle on right side

**Figure 2:**
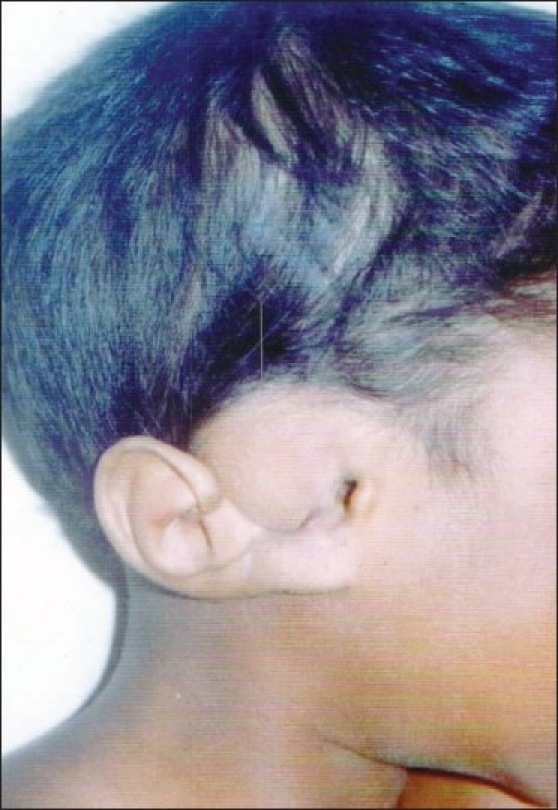
Profile view showing upper auricle attached to temple with ear lobule only on right side

**Figure 3:**
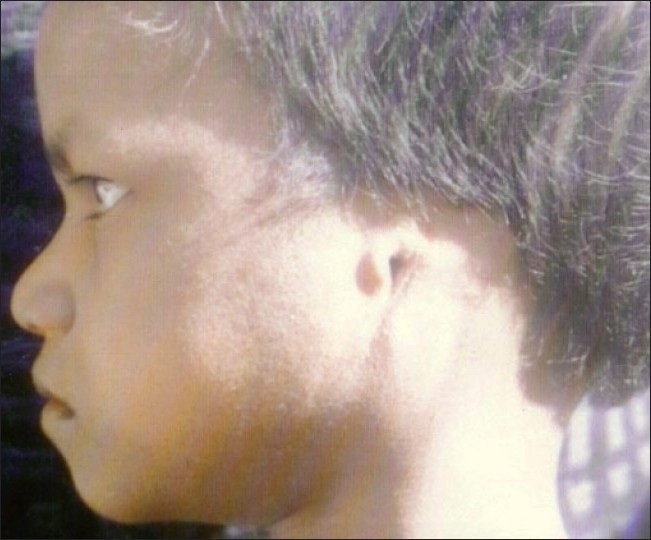
Profile view showing anotia on left side

On examination, the right auricle was normal in size and shape but hanging from the temple by ear lobule only [Figures 1, 2]. On the left side there was complete absence of external ear [Figure 3]. External auditory meatus and tragus were normal on both the sides.

### Case 2

**Figure 4:**
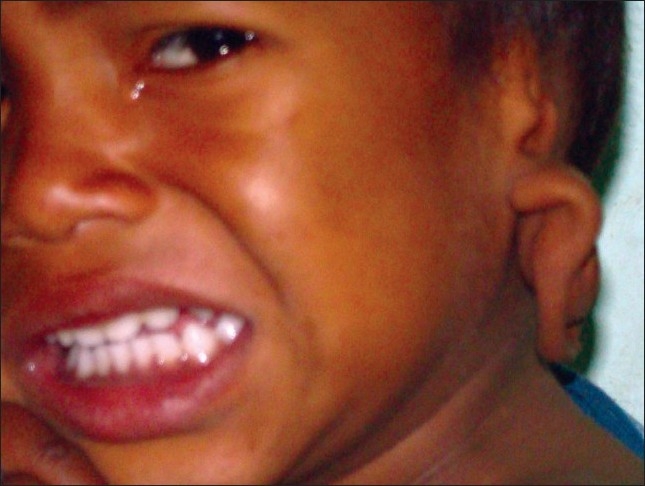
Frontal view showing upper auricular detachment with downward rotation of auricle on left side

**Figure 5:**
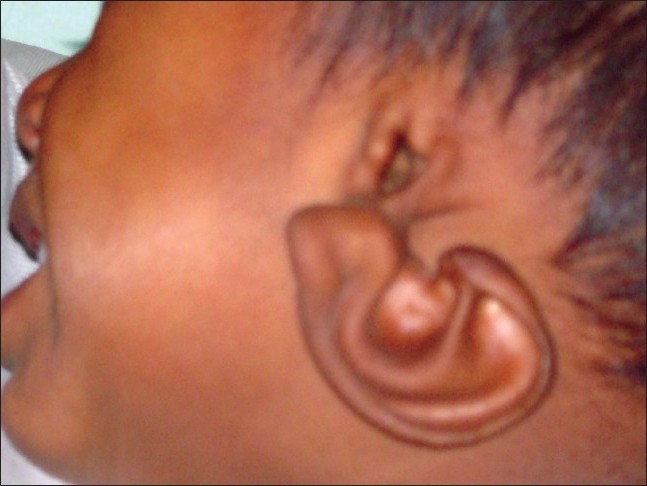
Profile view showing upper auricle attached to temple with ear lobule only on left side

A five-year-old boy presented with congenital anomaly of the left auricle. Antenatal history was uneventful and without any exposure to teratogenic agents. The child was full term normally delivered with no history of birth trauma. On examination, the left auricle was normal in size and shape but hanging from the temple by only the ear lobule [Figures 4, 5]. The right side ear was normal. External auditory meatus and tragus were normal on both the sides.

No other congenital anomalies were found in both the cases. A special attempt was made to survey all the other structures that develop from the 1^st^. and 2^nd^. Branchial arches, - the mandible, maxilla, zygoma and squamous part of temporal bone, the muscles of mastication and facial expression, the tongue, the parotid and the cranial nerves and clinical examination revealed no abnormality besides the auricular malformation.

**Figure 6:**
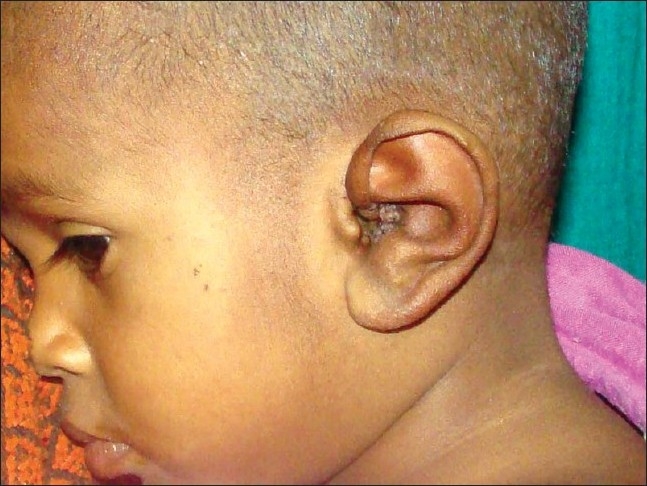
repositioning of auricle after surgical correction

For surgical correction, the desired position of ear was marked with ink on the temple. After intubation, a paring incision was made on the tragal side ear and a vertical incision was given on the temple on the premarked side; the ear was reattached to the temple by fixing the ear cartilage to the fascia over temple region with non absorbable sutures and skin incision closed [Figure 6].

## DISCUSSION

**Figure 7:**
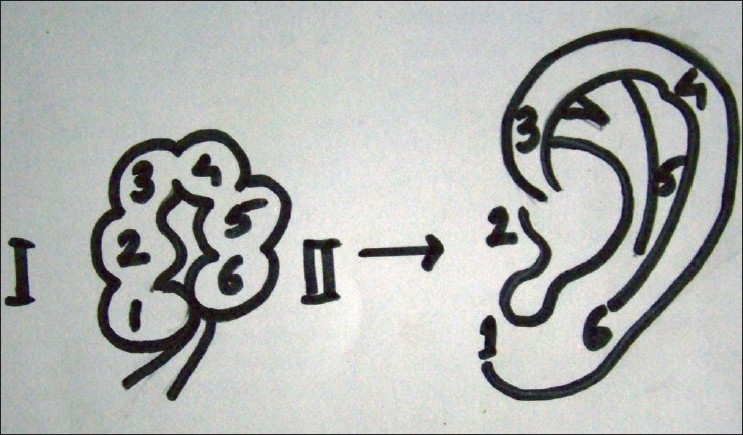
Six hillocks 1-3 on1st branchial arch and 4-6 on 2nd branchial arch

Congenital malformations of the pinna and external ear canal are related to the developmental defects of the first and second branchial arches and the branchial groove, which joins the first pharyngeal pouch to form the external ear canal. These malformations may occur singly or in combination. Embryologic and foetal development of the human ear is controversial. The pinna develops around the first branchial groove. Six hillocks appear on the first (mandibular) and second (hyoid) branchial arches in a 38 day-old embryo; these hillocks of His are numbered from 1 to 6; hillocks 1, 2 and 3 are derived from mandibular arch and hillocks 4, 5 and 6 are derived from hyoid arch [Figure 7]. These hillocks present on the facing border of each of the arches fuse to form the elevations, fossae and sulci of the adult pinna by the process of fusion and accretion. Since the process of fusion and accretion is complicated there is a controversy regarding contribution of each hillock in formation of different parts of ear. In general it is postulated that hillock1 and 6 produces the ear lobe, hillock 4 and 5 produces the antehelix or helix, hillock 2 produces the tragus and hillock 3 produces the ascending helix. The external auditory meatus is a derivative of the first ectodermal groove between the mandibular and hyoid arches.[2–4]

His reported that hillock 1 forms the tragus, hillock 2and 3 forms the helix, hillocks 4 and 5 forms the antehelix and scapha and hillock 6 forms the lobule.[2] Streeter reported that the tragus and the anterior crus of helix are derived from the mandibular arch, and the remaining helix, antehelix, scapha, antitragus and the lobule from the hyoid arch.[3]

Wood-Jones and Wen reported that only tragus is of mandibular arch derivative. They supported their theory by claiming that the line of election of preauricular fistula coincides with the line of the first pharyngeal depression and in cases of agnathia external ear were complete with the exception of tragus.[5]

In our case, only tragus remained on the mandibular arch side of the auricle suggesting that only tragus is a part of mandibular arch and the root of helix and ascending helix is the part of hyoid arch derivatives. Our case confirms the theory of Wood-Jones and Wen that the boundary of mandibular arch does not extend beyond the tragus and whole external ear is hyoid arch derivative except tragus.

This deformity may be due to the failure of mesenchymal fusion or accretion between the hillocks of mandibular and hyoid arches. Another explanation for such a deformity may be congenital amniotic band which cause disruption of auricular attachment, but this explanation is unlikely since there was no circumferential or hemi circumferential lesion was present.

## SUMMARY

Two unusual cases of congenital bilateral ear deformity have been presented. The deformity characterized by upper auricular detachment on the right side with anotia on the left side in first case and upper auricular detachment on the left side with normal ear on the right side in second case. An attempt has been made to correlate the presented deformity with the embryological – foetal development of the auricle. Satisfactory correction can be obtained by repositioning the auricle back in to its normal position.
